# Telemedicine for Optimizing Secondary Prevention in Coronary Artery Bypass Grafting Patients during COVID-19 Pandemic

**DOI:** 10.3390/healthcare11111590

**Published:** 2023-05-29

**Authors:** Luminița Iliuță, Andreea Gabriella Andronesi, Marius Rac-Albu, Mădălina-Elena Rac-Albu, Alexandru Scafa-Udriște, Horațiu Moldovan, Florentina Ligia Furtunescu, Bogdan Constantin Rădulescu, Eugenia Panaitescu

**Affiliations:** 1 Medical Informatics and Biostatistics Department, University of Medicine and Pharmacy “Carol Davila”, 050474 Bucharest, Romania; 2 Cardioclass Clinic for Cardiovascular Disease, 031125 Bucharest, Romania; 3 Nephrology Department, University of Medicine and Pharmacy “Carol Davila”, 050474 Bucharest, Romania; 4 Nephrology Department, Fundeni Clinical Institute, 022328 Bucharest, Romania; 5 Department of Cardio-Thoracic Pathology, University of Medicine and Pharmacy “Carol Davila”, 050474 Bucharest, Romania; 6 Department of Cardiovascular Surgery, Clinical Emergency Hospital, 014461 Bucharest, Romania; 7 Academy of Romanian Scientists (AOSR), 050045 Bucharest, Romania; 8 Department of Cardiology, Clinical Emergency Hospital, 014461 Bucharest, Romania; 9 Department of Public Health and Management, Faculty of Medicine, “Carol Davila” University of Medicine and Pharmacy, 050474 Bucharest, Romania; 10 C.C. Iliescu Emergency Institute for Cardiovascular Diseases, 022328 Bucharest, Romania

**Keywords:** telemedicine, cardiovascular prevention, teleprevention, coronary artery bypass grafting, remote monitoring, secondary prevention, cardiovascular risk, cholesterol, COVID-19

## Abstract

(1) Background: The COVID-19 pandemic has introduced a major disruption to the delivery of secondary prevention measures in patients with established atherosclerotic cardiovascular disease (CVD). It required a rapid and widespread adoption of new medical services, including the use of telemedicine. This study aimed to examine the impact of COVID-19 on secondary prevention in patients with coronary artery bypass grafting (CABG) and to evaluate the effectiveness of the telemedicine application for the implementation of lifestyle change measures, remote monitoring, and treatment regimen adjustment; (2) Methods: This prospective study on 194 CABG patients evaluated three consecutive years between 2019 and 2022 in the pre-pandemic period by face-to-face visits and during the pandemic by teleconsultations or hybrid follow-up. Variables of interest were compared between four periods: pre-pandemic—pre-P (1 March 2019–29 February 2020), lockdown—Lock (1 March–31 August 2020), restrictive-pandemic—Restr-P (1 September 2020–28 February 2021), and relaxed–pandemic—Rel-P (1 March 2021–1 March 2022). (3) Results: The average values of the lipidogram, blood sugar, and uric acid increased during Lock and Restr-P, but, through the use of teleprevention, they returned to the pre-pandemic level or even below this level. The exception was blood sugar, which remained high in Rel-P. The number of newly diagnosed patients with diabetes also increased, with most of them having moderate forms of COVID. During Lock and Res-P, the percentage of obese, smoking, or hypertensive patients increased, but, through the use of teleprevention, we managed to reduce it, although it remained slightly higher than the pre-pandemic level. Physical activity decreased in the first year of the pandemic, but, in Rel-P, CABG patients became more active than before the pandemic (4) Conclusions: The use of telemedicine for cardiovascular secondary prevention allowed us to not only continue seeing CABG patients but, also, to adjust their medication and to expand cardiovascular preventive counseling and testing with favorable results, especially during the second year of the pandemic.

## 1. Introduction

The 2019 coronavirus (COVID-19) pandemic created significant challenges for healthcare services and the cardiovascular patient community was one of the most exposed [1]. It forced the rapid adoption of best practices for caring for patients with COVID-19 and cardiovascular disease (CVD) and managing cardiovascular complications [2]. The COVID-19 pandemic also caused significant anxiety among patients with chronic CVD about COVID-19 and their condition. They faced the cancellation or postponement of scheduled consultations and investigations as well as delays in adjusting doses and prescribing drugs [3,4].

After the onset of the pandemic, the importance of cardiovascular prevention was overshadowed, and the focus of all cardiologists moved more toward reaction, intervention, and emergency treatment [5,6,7,8]. Regarding the monitoring of cardiovascular risk factors and secondary prevention, this was left last. Due to the special measures implemented during the COVID-19 pandemic, cardiovascular prevention and the implementation of a healthy lifestyle were neglected. Primary and secondary prevention was also significantly affected by changes in lifestyle and dietary behavior during social isolation. This increased consumption of processed foods, snacks, and alcohol and in sedentary behavior, with reduced physical activity resulting in weight gain and increased rates of social isolation, mental stress, depression, and loneliness [9]. In addition, the amplification of messages that those with chronic conditions should stay at home confused and scared patients with cardiovascular conditions, increasing anxiety levels. This led to a delay in the evaluation of symptoms that can hide the aggravation of underlying conditions resulting in delayed acute and preventive care [10].

In our country, the public health crisis caused by the COVID-19 pandemic has dramatically reduced cardiovascular prevention services, both primary and secondary, especially in the first period, as well as all measures to implement a healthy lifestyle. Patients with chronic coronary syndrome as well as those with coronary artery bypass grafting (CABG) represent a vulnerable population in which secondary prevention is very important since they are at high risk of the disease progressing if preventive measures are neglected.

On the other hand, the pandemic helped us find alternative methods, such as the development of telemedicine, to provide support and care to stable patients. Thus, new applications and platforms have been developed for doctor-patient interaction, improving both remote monitoring of chronic patients and preventive care [11,12,13,14].

The vast majority of patients were consulted through direct patient-doctor contact before the pandemic. However, telemedical management has become a major follow-up strategy for chronic patients with CVD to reduce the risk of COVID-19 in these vulnerable patients. Patients were enrolled (some even proactively) in telehealth programs for remote monitoring of the condition [12,13,14]. The telemedicine applications included targeted questions as well as recommendations regarding changes in diet, alcohol consumption, psychological health, or physical activity.

We immediately adapted our clinic-dedicated application by adding a telemedicine platform for the remote monitoring of chronic patients, with the adaptation of monitored parameters depending on the pathology, and for the implementation of primary and secondary prevention measures.

The first aim of our study was to analyze how the COVID-19 pandemic affected major cardiovascular risk factors and disrupted secondary prevention services and cardiovascular care in CABG patients.

In addition, we tried to evaluate the effectiveness of the telemedicine application for remote monitoring, adjustment of treatment regimens, and implementation of healthy lifestyle measures to improve remote cardiovascular prevention in CABG patients.

## 2. Materials and Methods

### 2.1. Study Population, Setting, and Data Collection

We performed a prospective study that included 194 patients with CABG recorded in the existing dedicated application of the Cardioclass Clinic for cardiovascular diseases before the beginning of the study. Patients were evaluated for three consecutive years between 1 March 2019 and 1 March 2022. The dedicated application was extended during the lockdown with a telemedicine module that provides remote monitoring of the patients. All patients included signed the informed consent form (which was approved by the institutional ethics committee, protocol code 306/17 February 2019) authorizing prospective data collection for research purposes. We also obtained the consent of the patients monitored through the telemedicine-added module regarding the processing of personal data.

Inclusion criteria: Patients were eligible for enrolment in the remote monitoring program if they had been evaluated in our clinic and registered in the dedicated application within the preceding 12 months before the beginning of the study.

Exclusion criteria:

- patients without access to a computer connected to the internet or a smartphone
- patients who were absent for more than 50% of the follow-up visits (face-to-face or teleconsultations)
- elderly patients who preferred to defer teleconsultations in favor of obtaining in-person visits

In the pre-pandemic period (from 1 March 2019 to 29 February 2020), standard patient follow-up consisted of in-person appointments (minimum of one appointment per trimester) with a physician consultation, electrocardiogram (ECG), echocardiographic examination, and/or ambulatory ECG or BP monitoring. We provided learning instruments to instruct the patients to follow the important parameters, these being evaluated by the attending physician at each visit, but we did not introduce them in the dedicated application before the pandemic because we added the telemedicine module for remote monitoring at the beginning of the lockdown period. Follow-up phone calls were scheduled to check patients’ symptoms, to monitor the need for drug prescription renewal, and to adjust drug doses (mainly for dyslipidemia, arterial hypertension, or antianginal medication).

During the first year of the pandemic, which included the lockdown period and the restricted pandemic period (from 1 March 2020 to 28 February 2021), face-to-face visits were drastically reduced (with no in-person appointments during the lockdown till 15 May 2020), being replaced by teleconsultations. In-person appointments were limited to one visit during the restricted pandemic period and to urgent/emergent situations when it was possible to perform other elective diagnostic and therapeutic procedures (transthoracic echocardiograms, ambulatory ECG, and BP monitoring). Due to COVID-19 constraints, we had to cancel all the stress tests (including the exercise stress test and stress echocardiography). All patients were remotely monitored using the multiparametric telemedicine module added to the dedicated application.

In the relaxed-pandemic period, under the umbrella of vaccination (from 1 March 2021 to 1 March 2022), we provided a hybrid follow-up of the patients with teleconsultations and remote monitoring using our dedicated multiparametric application and a minimum of two in-person appointments per year (including physician consultation, ECG, echocardiographic examination, and/or ambulatory ECG or BP monitoring).

During the study, all patients also had access to a dedicated phone number and a dedicated email to contact the team in case of any sign or symptom occurring. Patients used a self-monitoring registry of vital signs and symptoms. Teleconsultations and parameters collected using remote monitoring helped us to identify patients who needed in-person care and also to adjust the medication according to the symptoms or the laboratory parameters. The prescription of medicines was by e-mail or via the short message service. To check drug adherence, we monitored the need for drug prescription renewal using our dedicated application as well as telephone requests.

### 2.2. Parameters Monitored, Alerts, and Cardiovascular Risk Score

The dedicated application utilized all the clinical and paraclinical data of the patients who were evaluated in the clinic and signed the informed consent. The data included the following:

- history, cardiovascular risk factors;
- symptoms, clinical evaluation;
- the investigations carried out in the clinic—electrocardiogram (ECG), echocardiography, ambulatory blood pressure (BP) or ECG monitoring, stress test, and carotid or peripheral arterial Doppler ultrasound;
- medication; and
- blood tests.

The multiparametric module connected to our dedicated application was used during the pandemic for remote monitoring of all the patients included in the study. The telemedicine module allowed patients (using the application platform with a friendly interface) to transmit information about their BP, HR, weight, remote ECG (via an Istel HR-2000 6-lead ECG recorder with four built-in electrodes as a remote monitoring system—Diagnosis SA, Bialystok, Poland) and responses to questions relating to five coronary artery disease (CAD) symptoms to a remote monitoring server. In addition, at least twice yearly, for each patient included in the study, we collected information about the main monitored cardiovascular risk factors and blood tests (total cholesterol [TC], LDL cholesterol, HDL cholesterol, triglycerides [TGL], glucose, uric acid, urea, creatinine, transaminases, and glycated hemoglobin for diabetic patients). To optimize this out-of-hospital management of the CABG patients, many blood tests were made in local laboratories, and we also allowed home-based phlebotomy. All collected data were monitored by our specialized team of doctors and nurses trained to use the remote monitoring application.

To simplify the monitoring process and the alerts system, the responses to questions related to angina symptoms were classified as Good, Attention, and Alert, using an adapted Seattle Questionnaire [15], as it is presented in Supplementary Materials:

Using the values of the monitored parameters, five alerts were programmed in the monitoring application based on their critical absolute values or important changes over time. The main programmed alerts for the diagnosis of angina decompensation were as follows:

- chest pain, chest tightness, or angina more than 4 times/day;
- administration of nitrates (nitroglycerin tablets) more than 4 times/day due to chest pain, chest tightness, or angina;
- limitation for walking indoors on level ground or for dressing due to chest pain, chest tightness, or angina;
- mean HR of more than 100 b/min over 3 consecutive days or paroxysmal atrial fibrillation; and
- changing symptoms from Good to Attention or Alert from one week to another.

The cardiovascular risk score was calculated using the clinic’s dedicated application (in which all the clinic’s patients were enrolled before the beginning of the study), as the telemedicine module was connected to it. The application calculated the risk score at enrollment into the study as a sum of main risk factors (older age, male, family history of cardiovascular disease, arterial hypertension, high levels of low-density lipoprotein cholesterol or triglycerides, diabetes, smoking or secondhand smoke exposure, obesity, unhealthy diet, physical inactivity, and stress). At each visit, the dedicated application calculated the updated cardiovascular risk score as a sum of non-modifiable risk factors (older age, male, family history of cardiovascular disease) and modifiable risk factors. For calculation of the updated cardiovascular risk score, the application took into account a modifiable risk factor only when it was uncontrolled, either by changing life habits or by drug treatment (uncontrolled arterial hypertension, high level of LDL cholesterol or triglycerides corresponding to risk group, uncontrolled diabetes, smoking or passive smoking, obesity, unhealthy diet, physical inactivity, and stress). The maximum calculated score using the dedicated application algorithm is 10, and it has a good correlation with the SCORE2 new model of the European Society of Cardiology from 2021 (Pearson r = 0.72, *p* < 0.05) [16]. We used the risk score of our dedicated application to facilitate patient follow-up and to have a more detailed analysis of risk factors, as this calculation algorithm takes into account more risk factors than the classical scores used.

Also, depending on the correctable risk factors of each patient, we extended the telemedicine module by creating a special section of the telemedicine application to automate the process of correcting cardiovascular risk factors, customized according to the needs of each patient. It automatically transmitted via email personalized information with advice on diet and a physical activity program, thus trying to promote a healthy lifestyle.

### 2.3. Study Periods and Variables of Interest

Parameters monitored were compared between four periods:

- pre-pandemic—pre-P (1 March 2019–29 February 2020), with a duration of 1 year;
- lockdown—Lock (1 March–31 August 2020), with a duration of 6 months;
- restrictive-pandemic—Restr-P (1 September 2020–28 February 2021), with a duration of 6 months; and
- relaxed–pandemic—Rel-P (1 March 2021–1 March 2022), with a duration of 1 year.

We chose to extend the lockdown period to 6 months as long as the lockdown lasted in our country (imposed isolation with the state of national emergency was declared from 1 March to 16 May 2020), because, during that period, patients did not have access to analyses, many data were missing, and the results would have been inconclusive. During the restrictive pandemic, as in other countries, social distancing restrictions were imposed, which were relaxed during Rel-P under the covering umbrella of vaccination.

In the clinic’s dedicated application, all data resulting from face-to-face consultations were recorded (symptoms, clinical parameters, blood tests, ECG, and echocardiography) in addition to ambulatory BP and ECG monitoring and teleconsultations as well as updated medication at enrollment and during monitored periods for three years.

The telemedicine application was used to collect data weekly on BP, HR, weight, symptoms, ECG, and, at least twice yearly, on cardiovascular risk scores and blood tests. It recorded alerts and angina decompensation events, medication changes, teleconsultations, and details of admissions into the clinic for intravenous treatment. Angina decompensations were reported by the cardiologists from the remote monitoring center and resulted in an increase in antianginal treatment.

Also, we recorded the COVID-19 illnesses and their form (mild, medium, severe) as well as vaccinations (moment, type of vaccine, and the number of doses).

### 2.4. Statistical Analysis

Statistical analysis was performed using SPSS version 23.0. Quantitative data are reported as mean ± standard deviation (SD) and qualitative data are summarized as absolute values with corresponding percentages. The association between pre-pandemic and intra-pandemic parameters was based on the Pearson correlation analysis and linear regression analysis.

We used parametric or nonparametric paired tests to compare two time-point estimates (paired Student *t*-test, or Wilcoxon-rank test). To compare variables assessed at three different time points, we used a repeated-measures analysis of variance (ANOVA), after checking for normality and homoscedasticity (homogeneity of variances) with the conventional tests. When these assumptions were violated, we used the non-parametric repeated ANOVA (Friedman test). When the F-ratio of the ANOVA or the Friedman test reached a critical level (corresponding to a *p* < 0.05), post hoc analysis with P-value adjustment for multiple comparisons was used. Categorical paired nominal data at two time points were compared with the McNemar test.

We used a Pearson Chi-Square to analyze if there is a statistically significant difference in prevalence (binary outcome) between three or more than three groups. Parametric or nonparametric paired tests were used to compare two time-points estimates (paired Student *t*-test, or Wilcoxon-rank test) and baseline characteristics of the study groups (Chi-Square and ANOVA).

To check the conditions of the models, the values for asymmetry and kurtosis between −2 and +2 were considered acceptable to prove normal univariate distribution [17,18]. In addition, we considered normal distribution if skewness is between −2 and +2 and kurtosis is between −7 and +7 [19]. Mauchly’s test of sphericity was significant (*p* < 0.001) so the assumption of sphericity had not been met. We used the Greenhouse-Geisser correction when Epsilon was < 0.75 and the Huynh-Feldt correction when Epsilon was >0.75. All tests were performed two-sided and a *p*-value of <0.05 was considered statistically significant.

## 3. Results

All patients included in the study had chronic coronary syndrome with CABG in their personal medical history and were registered in the clinic’s dedicated application with all clinical parameters and laboratory investigations at least one month before the beginning of the study. Initially, we included 200 CABG patients but 6 were lost at the follow-up visits, so the analysis was conducted on the remaining 194 patients for whom we obtained complete data. Most of the patients were male (78.4%) and the mean age was 72.01 years (±11.07).

In addition, 52.58% of the patients also had associated percutaneous transluminal angioplasty in the past, and 42.28% of them had a myocardial infarction.

Regarding the main cardiovascular risk factors monitored, when enrolling into the study, 64.4% of patients had hypertension, 25.3% of patients had diabetes, 29.9% of patients were obese (BMI > 30 kg/m^2^), 11.3% were smokers, 35.6% were sedentary patients, 29.9% had chronic alcohol consumption (more than 80 ml alcohol intake daily) and 38.2% declared high levels of stress. The mean (SD) initial cardiovascular risk score at enrollment into the study was 5.96 (±2.027).

Taking into account comorbidities, 33.5% of the patients had at least one episode of paroxysmal or persistent atrial fibrillation, and 6.9% had a heart failure NYHA classification of more than 2. At enrollment in the study group, the average (SD) heart rate was 65b/min (±11.55), the mean (SD) systolic blood pressure was 143.39 mmHg (±19.58), and the mean (SD) diastolic blood pressure was 77.89 mmHg (±8.92).

The majority of patients with CABG received target doses of the standard medication for this disease in accordance with the current guidelines (lipid-lowering agents, beta-blockers, angiotensin-converting enzyme inhibitors, antiplatelets, nitrates, and anticoagulants when indicated). At the time of enrollment, treatment for dyslipidemia included statins in 93.3% of the patients (rosuvastatin 39.8%, atorvastatin 57.5%, and simvastatin 2.8%), fibrates (fenofibrate) in 17.0%, ezetimibe in 27.3%, and supplements (omega-3 fatty acids, monacolin, other supplements) in 30.4% of the patients.

The percentage of anti-COVID-vaccinated patients was 97.42% (189 pts). During the pandemic, there were 13 patients diagnosed with COVID-19 (6.7%), 11 patients with a mild form and favorable outcome (no cardiovascular or respiratory complications, with only mild symptoms, 72.72% of them being vaccinated), 2 patients with a moderate form requiring hospitalization (of whom no one was vaccinated), and no patients with severe disease.

The percentage of patients who reached the therapeutic targets according to the ESC guidelines [20] regarding LDL cholesterol and TGL increased significantly in Rel-P compared to the pre-pandemic period, highlighting the effectiveness of the telemedicine application in secondary teleprevention (Table 1). The percentage of patients with LDL cholesterol < 55 mg/dL was 5.7% before the pandemic, 3.9% during Lock, 8% during Restr-P, and an increase of more than two-fold during Rel-P (12.2%). The percentage of patients with uncontrolled dyslipidemia with LDL cholesterol > 100 mg/dL increased significantly during Lock and Restr-P (from 55–60% and decreased in Rel-P to almost half [27.9%]). The target TGL of <150 mg/dL was reached in 84.5% of the patients in Rel-P compared to only 40.6% during Restr-P. In addition, the number of newly diagnosed patients with diabetes mellitus increased significantly during the pandemic. Glucose > 120 mg/dL was found in 26% of patients in the pre-P, in 46.5% of patients during Lock, in 53.9% of patients during Restr-P, and almost three times higher during Rel-P (68.1%) compared to the prepandemic period.

Adherence to lipid-lowering medication decreased significantly during Lock. This effect was due to the restrictions imposed and having to stay mostly at home, so it was difficult for many patients to procure their medication. During Restr-P and Rel-P, to check drug adherence, we monitored the need for drug prescription renewal using our dedicated application and telephone requests. Consequently, treatment adherence increased significantly. Through the telemedicine application, we managed to increase the doses of lipid-lowering drugs to obtain better control (Table 2).

Participants have recorded blood tests for four time periods: Pre-P, during Lock, Restr-P, and Rel-P. Because of missing data in some periods and to provide a statistical analysis as robust as possible, we used the Paired Samples T Test (which considers only the number of pairs with values at the two time points). Normality checks were carried out on the residuals and original values. The normality tests show a significant difference, but the values for skewness and kurtosis are within acceptable limits. So, a comparison between the four analyzed periods (Pre-P with Lock, Restr-P, Rel-P, Lock with Restr-P, Rel-P, and Restr-P with Rel-P) using the Paired Samples T Test showed a significant increase of total-cholesterol, LDL cholesterol, and triglycerides (TGL) levels during Lock and Restr-P compared to Pre-P.

During the pandemic, the average values of TC, LDL cholesterol, triglycerides, and uric acid increased during Lock and Restr-P compared to Pre-P with statistically significant differences. During the Rel-P period, they decreased close to the baseline level.

A repeated measures ANOVA with a Greenhouse-Geisser correction showed that mean TC differed significantly between time points [F(1.774, 259.011) = 66.483, *p* < 0.001]. Post hoc tests using the Bonferroni correction revealed that TC increased compared to Pre-P by an average of 25.99 mg/dL after 1 year during Lock, by 28.22 mg/dL during Restr-P and dropped by 8.89 mg/dL after 3 years, compared to the baseline. In addition, TC increased by 34.88 mg/dL between Lock and Restr-P and decreased by 8.07 mg/dL between Lock and Rel-P, and by 37.11 mg/dL between Restr-P and Rel-P. There was a significant difference between each pair of time points (*p* = 0.000001).

Also, mean LDL-cholesterol values differed significantly between time points [F(2.214, 296.728) = 74.756, *p* = 0.000001]. LDL cholesterol increased compared to Pre-P by an average of 18.63 mg/dL after 1 year during Lock, by 19.88 mg/dL during Restr-P, and dropped by 5.43 mg/dL after 3 years compared to the baseline. In addition, LDL cholesterol increased by 2.74 mg/dL between Lock and Restr-P. It decreased by 24.06 mg/dL between Lock and Rel-P, and by 25.31 mg/dL between Restr-P and Rel-P. In addition, we found a significant difference between each pair of time points (*p* = 0.000001).

For HDL cholesterol values analysis, we used ANOVA repeated measures with a Huynh-Feldt correction. Mean HDL cholesterol differed significantly between time points [F(2.724, 378.671) = 96.004, *p* = 0.000001]. HDL cholesterol decreased compared to Pre-P by an average of 3.19 mg/dL after 1 year during Lock (*p* = 0. 0.000258), by 7.86 mg/dL during Restr-P (*p* = 0.000001) and increased by 5.26 mg/dL after 3 years (*p* = 0.000001) compared to the baseline. In addition, HDL cholesterol decreased by 4.66 mg/dL between Lock and Restr-P. It increased by 8.46 mg/dL between Lock and Rel-P, and by 13.12 mg/dL between Restr-P and Rel-P.

Also mean TGL differed significantly between time points [F(2.353, 327.029) = 57.389, *p* = 0.000001], increasing compared to Pre-P by an average of 32.37 mg/dL after 1 year, during Lock, by 46.72 mg/dL during Restr-P (*p* = 0.000001) and decreased by 1.76 mg/dL after 3 years (*p* = 0.000001) compared to the baseline. In addition, TGL increased by 13.80 mg/dL between Lock and Restr-P and decreased by 34.84 mg/dL between Lock and Rel-P, and by 13.80 mg/dL between Restr-P and Rel-P.

Summarizing, the trend evolution of the lipid profile showed a significant increase in the Pre-Lock period, followed by a slight increase in the Lock-Restr-P period and a reduction in the Restr-P-Rel-P period, sometimes even to a lower level than before the pandemic for TC, LDL-C, and TGL (Table 1).

The blood glucose level registered a different evolution curve. A repeated measures ANOVA with a Greenhouse-Geisser correction showed that mean glucose differed significantly between time points [F(1.749, 241.337) = 73.257, *p* < 0.001]. Mean glucose level increased compared to Pre-P by an average of 12.73 mg/dL after 1 year during Lock, by 16.63 mg/dL during Restr-P, and by 28.47 mg/dL after 3 years compared to the baseline. In addition, glucose levels increased constantly by 3.90 mg/dL between Lock and Restr-P, by 15.74 mg/dL between Lock and Rel-P, and by 11.84 mg/dL between Restr-P and Rel-P. There was a significant difference between each pair of time points (*p* = 0.000001).

The blood glucose values, however, increased throughout the pandemic (Table 1), with the highest increase in the Pre-P-Rel-P period, followed by Pre-P-Restr-P and Lock-Rel-P. During the Rel-P period, the number of patients with newly discovered DM increased significantly compared to previous periods and 79.5% of them had mild/moderate forms of COVID-19, which is consistent with the results of other studies that showed that COVID-19 is associated with aberrant glucose control, which can persist even after recovery [21]. The univariate regression analysis showed a statistically significant correlation between the mild or moderate form of COVID-19 infection and blood sugar levels > 110 mg/dL (r = 0.79 Pearson, *p* < 0.005).

**Figure 1:**
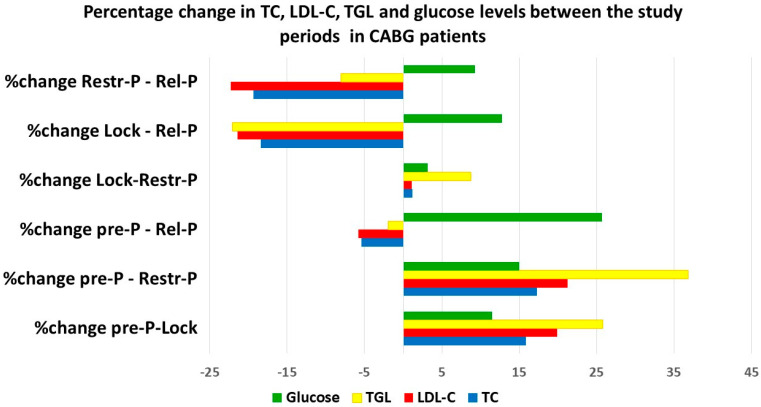
The evolution (percentage change between analyzed periods) of TC, LDL-C, HDL-C, TGL, and glucose levels during the pandemic.

The analysis of the percentages of change of the monitored parameters in the analyzed periods (Figure 1) showed the greatest increase for TGL between the Pre-P and Restr-P periods, followed by blood glucose between Pre-P and Rel-P. Regarding LDL-C and TC, they did not suffer such large increases compared to the pre-pandemic level as did TGL. On the other hand, looking at the percentage reduction between the studied periods, the most important reduction was recorded in LDL-C between the Restr-P and Rel-P periods, followed by the Lock-Rel-P period, when TGL also recorded a significant percentage reduction.

Regarding the cardiovascular risk score and the other modifiable cardiovascular risk factors, they also had an increasing trend during the pandemic, as illustrated in Table 3. The highest growth trend was recorded in the number of patients diagnosed with diabetes, which increased significantly throughout the pandemic. Medium BMI increased during the pandemic and the percentage of obese persons increased significantly during Lock. In terms of physical activity, the percentage of inactive people increased during the pandemic, but at the end of the pandemic, it decreased significantly compared to the pre-pandemic period.

The percentage of patients with hypertension and obesity increased during the restricted pandemic, but by using the telemedicine application and the increase in medication, we managed to reduce it. However, it remained higher than the pre-pandemic level (67.5% of patients with hypertension in Rel-P vs. 64.4% in Pre-P, 40.2% of obese patients in Rel-P vs. 29.9% in Pre-P). On the other hand, although the rest of the modifiable risk factors (smoking, unhealthy diet, and alcohol consumption) increased significantly during Lock, in Rel-P, they decreased significantly, with most patients adopting healthier lifestyle habits than before the pandemic. Physical activity decreased in the first year of the pandemic, but in Rel-P, people became more active than before the pandemic.

In the pre-pandemic period, we noted a mean of 10 phone calls monthly with a dedicated nurse vs. a mean of 25 calls during the pandemic. All of the pre-pandemic appointments were face-to-face and included an echocardiographic evaluation. During the pandemic period, more than 80% of the appointments were conducted online. The percentage of patients with in-person appointments significantly increased by the end of the pandemic period (March 2021–March 2022), compared to 2020–2021. We were able to successfully titrate antianginal, hypertension, and dyslipidemia medications, even by online or phone appointments with close monitoring of ambulatory BP, HR, weight, symptoms, and blood test results, which were entered by the patients in the dedicated platform. We did not record an increased rate of in-clinic admissions for intravenous treatment (antianginal, antihypertensive, or antiarrhythmic) or adverse drug events during the remote follow-up.

In hypertensive patients, during the two pandemic years (including the lockdown), the elevated BP values were managed at home with an increase in antihypertensive drugs, taking into account the trends in systolic blood pressure (from home monitoring system).

The percentage of patients with paroxysmal atrial fibrillation increased during Lock (40.2%) and Restr-P (35.6%) compared to Pre-P (33.5%), but decreased in Rel-P (34%), being similar to the one before the pandemic. Most of them were managed at home using the telemedicine platform and teleconsultations.

In light of an increase in remote management of this cohort of CABG patients, we were able to maintain a low rate of admissions due to angina decompensation. Thus, regarding hospitalizations and emergency department (ED) visits due to angina decompensation, there was no statistically significant difference between the pre-pandemic and the pandemic period (angina hospitalizations—*p* = 0.73; ED visits *p* = 0.37). Furthermore, during the pre-pandemic period, there were 9 patients hospitalized and 19 patients treated in the ED due to angina exacerbation, compared with 8 patients hospitalized and 7 patients treated in the ED during the pandemic period.

The finding that the telemedicine application was effective and safe in terms of controlling blood pressure values, cholesterol, and secondary prevention in general, as an approach based on clinical practice, is reassuring. Blood pressure and cholesterol control via telemedicine may be a better option for some people but not for others, resulting in effective heterogeneity between study groups.

Overall, the clinical condition of CABG patients under remote multiparametric monitoring was minimally affected by the lockdown restrictions, despite a marked decrease in conventional healthcare measures. Regarding the results obtained through using this remote monitoring strategy combined with measures to educate patients and increase their adherence, we encourage the incremental use of telemedicine in CABG patients in the context of this or future pandemics and also in situations in which physical consultation might not be possible due to logistic issues. Future studies are needed to evaluate the long-term clinical impacts of telemedicine as well as to continue to improve and standardize telemedicine technology.

## 4. Discussion

Throughout the pandemic period, the main problem of the medical system was the concentration of medical activity on the treatment of patients affected by COVID-19, allocating massive medical and financial resources in this direction. Thus, patients with chronic diseases or patients with other pathologies were automatically placed on the second level of the medical service [7,22,23].

For patients with CVD, the pandemic was a very difficult period, with a significant reduction in face-to-face cardiology visits, delays in updating the therapeutic scheme and obtaining treatment, and placing secondary prevention measures in second place. Since interaction with hospitals was more difficult (only in cases considered urgent), it reached the situation where patients arrived very late to the cardiologist in situations of myocardial infarction, with studies showing more than a three-fold increase in time from the onset of symptoms to revascularization [24,25].

Cardiac preventive programs were interrupted during the pandemic, creating the conditions for an increase in cases of cardiac pathology [5,6,7,8,9]. Studies have shown a reduction in primary care visits and cardiovascular screening visits (with BP measurement, cholesterol level, and cardiovascular risk score assessment). In addition, cardiac rehabilitation programs were suddenly interrupted for CABG patients, leaving them without access to this resource which could reduce total and cardiovascular mortality by up to 30% compared with standard treatment [26].

On the other hand, looking back, the pandemic brought new platforms for interacting with patients eager to engage, presenting a unique opportunity to reset how we approach preventive care. One of the many lessons the pandemic has taught us regarding the care of patients with CVD is how quickly the field of telemedicine has advanced in response to such challenging circumstances [11,12,27,28,29]. The overnight adoption of telemedicine applications for monitoring patients with CVD, but also for preventive cardiology, is the most obvious example. Thus, although the number of visits to the doctor decreased significantly, the frequency of BP measurements and adjustment of the treatment regimen did not undergo important changes (patients being enrolled in various telemedicine programs), which highlights the potential of these innovative programs [30,31,32].

We examined the impact of the COVID-19 pandemic on the major cardiovascular risk factors and secondary cardiovascular prevention of patients with CABG under multiparametric remote monitoring. First of all, as other studies have shown, the number of face-to-face consultations was significantly reduced in the first year of the pandemic, for all cardiovascular risk groups, which could favor the decompensation of the patients and a significant increase in the cardiovascular risk score. However, despite the healthcare delivery barriers created by the COVID-19 pandemic, our study showed that the use of telemedicine allowed us to not only continue seeing patients with CABG but also to expand counseling and testing. Our medical team quickly adapted to the new conditions, managing to offer patients remote medical services and update appropriate treatments depending on the patient’s pathology.

During the COVID-19 pandemic, as other studies showed, we could observe the harmful effects of inactivity, lack of a proper diet, lack of sleep, prolonged exposure to TV shows, social distancing, and lack of preventive measures. Thus, all the major cardiovascular risk factors had a significant unfavorable evolution during the first year of the pandemic, significantly increasing the risk of cardiovascular diseases. In the case of CABG patients and chronic coronary syndrome, all these changes in the cardiovascular risk score increased the risk of myocardial infarction or alteration of grafts or native vessels with the need for reintervention. That is why we must emphasize the efforts to prevent cardiovascular diseases by all possible means, which also means the use of telemedicine for this purpose even in normal life conditions unaffected by the pandemic. We used all available methods for monitoring patients, using online technology for detecting cardiovascular risk factors and counteracting them in real time by changing diets or monitoring the level of physical activities. In addition, the application allowed us to change drug treatments in real time depending on the changes in the patient’s life as well as monitor the main hemodynamic parameters and symptoms to identify patients who needed an in-person cardiology visit. Using the telemedicine application, we successfully adapted the statin, ezetimibe and fibrate doses according to the lipidogram level without the need for an in-person consultation. By increasing the number of teleconsultations, we managed patients’ symptoms and hypertension without the need for exposure to the hospital environment.

Thus, by keeping patients in permanent contact with our medical team and offering opportunities to reduce their cardiovascular risk and improve their lifestyle, towards the end of the pandemic, the patients enrolled in the study became more active, quit smoking, and improved their eating habits.

On the other hand, blood sugar increased constantly during the pandemic, especially during Lock and Restr-P. An explanation could be the fact that among the newly diagnosed cases of DM, the percentage of patients with a COVID-19 infection was higher. As other studies showed, COVID-19 is associated with aberrant glucose control, which can persist even after recovery [21]. We also found a good correlation between the infection with COVID and newly discovered DM after the pandemic.

On the other hand, it is difficult to explain the deterioration of glycemic levels in the Relax-pandemic period by the lack of exercise or excessive caloric intake or only by the presence of a COVID-19 infection. The number of patients diagnosed with DM increased significantly during the pandemic and our attached telemedicine module was managed by cardiologists who had in mind cardiovascular-specific medication. Access to a diabetologist was difficult due to the pandemic and the fact that many of the patients with newly diagnosed diabetes took hypoglycemic medication late and adjusted their diet late with the help of a nutritionist may explain the fact that the blood sugar level increased even in Rel-P. In our country, patients’ access to a diabetologist is through the family doctor. Because the interoperability between the various existing applications is deficient, there are usually delays when a patient needs to be picked up and treated in the diabetes-nutrition network, and this was even more difficult during the pandemic. For this reason, there was a latency period between the time of diabetes diagnosis and the time of the patient’s registration in the diabetes network, with the prescription of specific medication and the correct diet. The teleprevention application facilitated the correct and prompt treatment of dyslipidemia and, probably for this reason, there were also inconsistencies between the level of triglycerides (which decreased in Rel-P) and blood glucose (which registered an increase in Rel-P). The increase in doses of fibrates and lipid-lowering medication can explain the reduction in triglyceride levels, although blood sugar had an increasing trend.

Thus, globally, the secondary remote prevention measures for CABG patients gave very good results, with their cardiovascular risk score being smaller in the Rel-P than before the pandemic, which is in agreement with the results of other studies evaluating telemedicine applications [33,34].

Although the highest percentage reductions in the lipidogram parameters were recorded during the Restr-P-Rel-P and Lock-Rel-P periods, they are not representative in terms of the effectiveness of the telemedicine application considering the context of the pandemic that disrupted lives. However, between pre-P (when all consultations were face-to-face) and Rel-P (when patient follow-up was hybrid, with the use of the teleprevention module), all parameters of the lipidogram were reduced. This is an argument in favor of the telemedicine application, but in order to draw a relevant conclusion, since our study for perfecting the telemedicine application is still ongoing, we will compare the pre-pandemic period with the post-pandemic period (1 March 2022–1 March 2023) because we do not know how relevant a study conducted in a period of crisis can be for testing effectiveness.

On the other hand, an advantage of using the telemedicine application in our study is the exercise of educating patients regarding the self-assessment of a patient’s state of health at a given moment, the awareness of the occurrence of some medical problems, and their monitoring. Patients were able to recognize new symptoms in real time and to report information about changes that occurred in their lifestyle so that doctors could change medical treatments or adjust treatment doses. Under the guidance of doctors, patients could change their unhealthy eating habits or engage in regular controlled physical activity, all of which ultimately lead to better health. [35,36].

The COVID-19 pandemic has also brought positive aspects to the lives of patients and to attending physicians through the use of these remote monitoring methods [37,38,39,40,41,42].

Our study is the first one to analyze the impact of telemedicine on cardiovascular counseling and testing of CABG patients during the COVID-19 pandemic in our country. We tried to highlight what the pandemic has taught us about caring for vulnerable CABG patients since they continue to need secondary prevention. Telemedicine was a life-saving solution for some of the patients, and our results support the ongoing use of telemedicine as a means to improve patients’ access to cardiovascular secondary prevention services.

In our study, the number of hospitalizations and emergency visits due to angina decompensation was not affected by the pandemic, which proves that the method used was safe and effective. The clinical status of CABG patients under multiparametric remote monitoring was minimally affected by pandemic restrictions despite a marked decrease in conventional measures of healthcare use.

This study presents a viable starting solution that can be developed and updated constantly. These already existing remote communication channels can provide complementary medical services to classic medical services, being easier to access in certain situations (patients with reduced mobility or living at great distances from medical facilities), not only during a pandemic. Our study demonstrated the potential of telemedicine to expand the ambulatory monitoring and secondary prevention of CABG patients.

### 4.1. Limitations of the Study

There are some limits to our study: it is a single-center study, with a small sample size, a short period of follow-up during the pandemic compared with the pre-pandemic period, and a low event number. However, we evaluated a large number of parameters with few missing data.

Our study highlighted some of the limitations of using telemedicine. During an in-person patient visit, it is very easy to obtain a patient’s blood sample. However, 14% of patients failed to go to the testing laboratory, generating missing data. Another difficulty was the fact that despite their higher risk for COVID-19, several older patients preferred to defer teleconsultations visits in favor of in-person visits, probably due to a lower comfort level with telemedicine technology in older adults. We were also unable to perform physical examinations via telemedicine, and also, we have no information regarding the specific technical problems that could have arisen during teleconsultations.

Our study did not address the level of satisfaction for patients or clinicians with the telemedicine application format, which represents an important aspect that should be analyzed by future qualitative studies, as telemedicine continues to develop.

Because of the interoperability problems encountered in our country with patients’ medical records, the electronic registries were not systematized and there is no software that may generate an alert email every time one of the CVD patients is admitted to the hospital. In addition, the access of patients with newly discovered or decompensated diabetes to the diabetologist was delayed due to the restrictions of the pandemic and the absence of telemedicine applications for the management of these patients. For this reason, glycemic control was achieved with a delay and its level remained high even in Rel-P.

### 4.2. Perspectives

Our results are based on the information obtained at a certain moment of the health crisis and do not represent the general experience in the practice of cardiovascular secondary prevention. Further research is needed to evaluate the perspectives regarding cardiovascular secondary teleprevention and the implications of using it on a large scale and for a more sustained period as well as in a non-pandemic context. Widespread use of telemedicine consultations remains a challenge due to technology, communication, education, and funding barriers.

Our telemedicine application is still under evaluation, but our findings suggest that a combination of home remote monitoring with less frequent in-person visits and teleprevention represents a good method for preserving the care and safety of CABG patients. Therefore, our experience argues for the benefits of this strategy in this patient population and supports larger studies in a non-pandemic context to confirm our findings.

## 5. Conclusions

Lipid levels increased significantly during the pandemic in the first year, but after that, by enrolling patients in telemonitoring, using the secondary prevention measures through the telemedicine application, and increasing medication, we managed to lower TC, LDL-C, and TGL, even to a lower level than the pre-pandemic one.

However, the blood glucose level increased constantly during the pandemic, parallel to the increase in the number of newly diagnosed cases of diabetes, which were reported especially in patients with a COVID-19 infection. The high glycemic level in Rel-P was due to delayed access to the diabetologist.

The main modifiable cardiovascular risk factors (hypertension, obesity, smoking, physical inactivity, and unhealthy diet) showed an increasing trend during the first pandemic year, but, through teleprevention measures and increased medication, we managed to reduce them, even if, some of them remained higher than the pre-pandemic level (hypertension, obesity, and declared level of stress). However, smoking, physical inactivity, and unhealthy diet had a favorable evolution. The percentage of patients with unhealthy lifestyles increased during the pandemic but significantly reduced at the end of the pandemic compared to the pre-pandemic period.

The telemedicine application for secondary cardiovascular prevention in CABG patients was effective, especially during the second year, after the learning curve was overcome. The patients were more receptive and the stress generated by the pandemic was reduced.

An increase in the use of a range of eHealth platforms has the potential to transform secondary prevention. Further studies and integrating research programs to evaluate the utility of these eHealth platforms may provide important information regarding the development of optimal applications for secondary cardiovascular prevention beyond the pandemic.

## Figures and Tables

**Table 1:** The evolution of lipidogram and glucose levels in the analyzed periods, depending on target limits according to 2019 ESC guidelines.

Variables	Pre-Pandemic (Pre-P) 1 March 2019–29 Feb 2020	Lockdown (Lock) 1 March 2020–31 Aug 2020	Restricted-Pandemic (Restr-P) 1 Sept 2020–28 Feb 2021	Relaxed-Pandemic (Rel-P) 1 March 2021–1 March 2022	*p* Value (ANOVA, Pearson Chi-Square)
TC (mg/dL), mean (SD)	N = 163 161.76 (41.031)	N = 158 187.66 (46.254)	N = 159 190.41 (54.872)	N = 150 154.83 (39.701)	0.001
TC > 190 mg/dL, no. (%)	30/163 (18.4%)	70/158 (44.3%)	69/159 (43.4%)	21/150 (14%)	0.001
LDL-C (mg/dL), mean (SD)	N = 159 96.67 (34.675)	N = 154 111.9 (36.777)	N=150 111.53 (41.819)	N = 147 90.13 (34.142)	0.003
LDL-C < 55 mg/dL, no. (%)	9/159 (5.7%)	6/154 (3.9%)	12/150 (8.0%)	18/147 (12.2%)	0.001
LDL-C 55–70 mg/dL, no. (%)	23/159 (14.6%)	9/154 (5.8%)	13/150 (8.7%)	22/147 (15.0%)	0.001
LDL-C 70–100 mg/dL, no. (%)	68/159 (43.0%)	49/154 (31.8%)	40/150 (26.7%)	66/147 (44.9%)	0.053
LDL-C > 100 mg/dL, no. (%)	58/159 (36.7%)	90/154 (58.4%)	85/150 (56.7%)	41/147 (27.9%)	0.003
HDL-C (mg/dL), mean (SD)	N = 165 47.06 (10.263)	N = 154 44.20 (14.024)	N = 153 39.48 (13.707)	N = 152 52.61 (13.822)	0.001
HDL-C > 60 mg/dL, no. (%)	29/165 (17.6%)	26/154 (16.9%)	15/153 (9.8%)	38/152 (25.0%)	0.431
HDL-C 40–60 mg/dL, no. (%)	93/165 (56.4%)	55/154 (35.7%)	55/153 (35.9%)	90/152 (59.2%)	0.001
HDL-C < 40 mg/dL, no. (%)	43/165 (26.1%)	73/154 (47.4%)	83/153 (54.2%)	24/152 (15.8%)	0.001
TGL (mg/dL), mean (SD)	N = 164 132.08 (128.496)	N = 158 158.79 (84.891)	N = 155 172.62 (50.909)	N = 125 121.91 (60.813)	0.001
TGL < 150 mg/dL, no. (%)	124/164 (75.6%)	98/158 (62.0%)	63/155 (40.6%)	125/148 (84.5%)	0.001
Glucose (mg/dL), mean (SD)	N = 169 112.67 (35.739)	N = 155 124.82 (33.666)	N = 154 129.40 (41.386)	N = 144 138.03 (36.838)	0.001
Glucose > 120 mg/dL, no. (%)	44/169 (26.0%)	72/155 (46.5%)	83/154 (53.9%)	98/144 (68.1%)	0.490
Glucose 100–120 mg/dL, no. (%)	50/169 (29.6%)	50/155 (32.3%)	43/154 (27.9%)	23/144 (16.0%)	0.001
Glucose < 100 mg/dL, no. (%)	75/169 (44.4%)	33/155 (21.3%)	28/154 (18.2%)	23/144 (16.0%)	0.001

TC—Total cholesterol; LDL-C—LDL-cholesterol; HDL-C—HDL-cholesterol; TGL—triglycerides.

**Table 2:** Lipid-lowering medication, medium doses, and adherence to treatment in the study periods in CABG patients.

Medication, No. (%)	Pre-Pandemic (Pre-P) 1 March 2019–29 Feb 2020	Lockdown (Lock) 1 March 2020–31 Aug 2020	Restricted-Pandemic (Restr-P) 1 Sept 2020–28 Feb 2021	Relaxed-Pandemic (Rel-P) 1 March 2021–1 March 2022	*p* Value (ANOVA, Pearson Chi-Square)
Statins, no. (%)—total	181 (93.3%)	101 (52.1%)	141 (72.7%)	182 (93.8%)	0.001
Rosuvastatin, no. (%)	72 (39.8%)	36 (19.9%)	58 (32.1%)	73 (40.3%)	0.001
Rosuvastatin, mean (SD) dose	10.07 (5.963)	10.58 (6.832)	13.59 (7.153)	15.06 (4.905)	0.003
Atorvastatin, no. (%)	104 (57.5%)	64 (35.4%)	81 (44.7%)	104 (57.5%)	0.041
Atorvastatin mean (SD) dose	25.31 (15.087)	25.22 (15.980)	28.11 (11.155)	32.86 (13.093)	0.041
Simvastatin, no. (%)	5 (2.8%)	1 (0.5%)	2 (1.1%)	5 (2.7%)	0.003
Simvastatin mean (SD) dose	14.00 (5.477)	12.50 (5.000)	15.00 (7.071)	15.00 (7.071)	0.003
Fenofibrate, no. (%)	33 (17.0%)	19 (9.8%)	25 (12.9%)	58 (29.9%)	0.003
Fenofibrate, mean (SD) dose	153.64 (7.528)	154.47 (7.434)	155.50 (7.783)	156.50 (8.216)	0.001
Ezetimibe, no. (%)	53 (27.3%)	34 (17.5%)	54 (27.8%)	114 (58.7%)	0.001
Supplements (Omega 3, monacolin), no. (%)	59 (30.4%)	44 (22.7%)	45 (23.2%)	50 (25.8%)	0.051

**Table 3:** The cardiovascular risk score and modifiable cardiovascular risk factors in CABG patients in the studied periods.

Variables	Pre-Pandemic (Pre-P) 1 March 2019–29 Feb 2020	Lockdown (Lock) 1 March 2020–31 Aug 2020	Restricted-Pandemic (Restr-P) 1 Sept 2020–28 Feb 2021	Relaxed-Pandemic (Rel-P) 1 March 2021–1 March 2022	*p* Value (ANOVA, Pearson Chi-Square)
Mean (SD) cardiovascular risk score	5.96 (2.027)	6.92 (1.765)	6.23 (1.658)	5.22 (1.652)	0.001
Cardiovascular risk score > 8, no (%)	20 (10.3%)	68 (35.1%)	43 (22.2%)	17 (8.8%)	0.001
Cardiovascular risk score > 5, no (%)	111 (57.2%)	168 (86.6%)	123 (63.4%)	100 (51.5%)	0.001
Arterial hypertension (systolic blood pressure > 140 mmHg or diastolic blood pressure > 90 mmHg)	125 (64.4%)	167 (86.1%)	134 (69.1%)	131 (67.5%)	0.001
Diabetes mellitus, no. (%)	49 (25.3%)	57 (29.4%)	65 (33.5%)	66 (34%)	0.003
Smokers, no. (%)	22 (11.3%)	43 (22.16%)	29 (14.9%)	16 (8.2%)	0.041
BMI	28.92 ± 19.525	29.61 ± 12.614	30.33 ± 8.751	28.36 ± 7.810	0.041
Obesity (BMI > 30), no. (%)	58 (29.9%)	79 (40.7%)	99 (51%)	78 (40.2%)	0.003
Physical inactivity	69 (35.67%)	143 (73.7%)	97 (50.4%)	52 (26.8%)	0.003
Unhealthy diet	45 (23.2%)	127 (65.5%)	67 (34.5%)	42 (21.6%)	0.003
Alcohol consumption	58 (29.9%)	120 (61.9%)	102 (52.6%)	55 (28.3%)	0.001
Declared high level of stress	74 (38.2%)	178 (91.7%)	180 (92.8%)	102 (52.6%)	0.001
Paroxysmal Atrial fibrillation, no (%)	65 (33.5%)	78 (40.2%)	69 (35.6%)	66 (34%)	0.051
Heart Failure (%)	6.7%	5.1%	5.7%	5.7%	0.051

BMI—body mass index.

## Data Availability

All data generated or analyzed during this study are included in this published article.

## Supplementary Materials

The following supporting information can be downloaded at: https://www.mdpi.com/article/10.3390/healthcare11111590/s1.
