# Supplementary material for: Telemedicine for Optimizing Secondary Prevention in Coronary Artery Bypass Grafting Patients during COVID-19 Pandemic
Source: Healthcare (Basel). 2023 May 29;11(11):1590. doi: 10.3390/healthcare11111590 (PMC10252220; doi:10.3390/healthcare11111590)
Supplement: Supplementary file 1 [file healthcare-11-01590-s001.zip › healthcare-2357707-supplementary.pdf]

Supplementary Materials: Adapted Seattle Questionnaire for classification of angina symptoms

Good—No chest pain at rest and during daily activities; rarely chest pain at the most intense level of activity

- No nitroglycerin for chest pain, tightness or angina until the last visit
- Daily activities without restrictions
- Without affecting the enjoyment of life due to chest pain
- No symptoms of depression, you have a zest for life

Attention— Chest pain, tightness or angina 3 or more times per week, but not every day

- Administration of nitroglycerin for chest pain, chest tightness or angina 3 or more times per week, but not every day
- Daily activities are moderately limited due to chest pain- limitation for running/jogging, lifting or moving heavy objects, participating in vigorous sports (e.g., swimming, tennis)
- Slightly limitation of the enjoyment of life due to chest pain
- Mild symptoms of depression, you feel more depressed than usual

Alarm— Chest pain, pressure or tightness more than 1 time/day

- Administration of nitroglycerin for chest pain, chest tightness or angina daily
- Daily activities are severely limited due to chest pain (limitation for dressing yourself, walking indoors on level ground, showering)
- Severe limitation of the enjoyment of life due to chest pain
- You feel very depressed, with the constant fear that you may have a heart attack or die suddenly.
